# The iPrevent Online Breast Cancer Risk Assessment and Risk Management Tool: Usability and Acceptability Testing

**DOI:** 10.2196/formative.9935

**Published:** 2018-11-07

**Authors:** Louisa L Lo, Ian M Collins, Mathias Bressel, Phyllis Butow, Jon Emery, Louise Keogh, Prue Weideman, Emma Steel, John L Hopper, Alison H Trainer, Gregory B Mann, Adrian Bickerstaffe, Antonis C Antoniou, Jack Cuzick, Kelly-Anne Phillips

**Affiliations:** 1 Department of Medical Oncology Peter MacCallum Cancer Centre Victoria Australia; 2 School of Medicine Deakin University Geelong Australia; 3 Centre for Biostatistics and Clinical Trials Peter MacCallum Cancer Centre Melbourne Australia; 4 Centre for Medical Psychology & Evidence-Based Decision-Making University of Sydney Sydney Australia; 5 Department of General Practice and the Centre for Cancer Research The University of Melbourne Melbourne Australia; 6 School of Primary, Aboriginal and Rural Health Care University of Western Australia Perth Australia; 7 Centre for Health Equity Melbourne School of Population and Global Health The University of Melbourne Melbourne Australia; 8 Centre for Epidemiology and Biostatistics Melbourne School of Population and Global Health The University of Melbourne Melbourne Australia; 9 Parkville Familial Cancer Centre Peter MacCallum Cancer Centre Melbourne Australia; 10 The Sir Peter MacCallum Department of Oncology The University of Melbourne Parkville Australia; 11 Department of Surgery The University of Melbourne Melbourne Australia; 12 Centre for Cancer Genetic Epidemiology Department of Public Health and Primary Care University of Cambridge Cambridge United Kingdom; 13 Centre for Cancer Prevention Wolfson Institute of Preventive Medicine Queen Mary University of London London United Kingdom

**Keywords:** clinical decision support, breast cancer, BRCA1 gene, BRCA2 gene, risk, preventive health, screening

## Abstract

**Background:**

iPrevent estimates breast cancer (BC) risk and provides tailored risk management information.

**Objective:**

The objective of this study was to assess the usability and acceptability of the iPrevent prototype.

**Methods:**

Clinicians were eligible for participation in the study if they worked in primary care, breast surgery, or genetics clinics. Female patients aged 18-70 years with no personal cancer history were eligible. Clinicians were first familiarized with iPrevent using hypothetical paper-based cases and then actor scenarios; subsequently, they used iPrevent with their patients. Clinicians and patients completed the System Usability Scale (SUS) and an Acceptability questionnaire 2 weeks after using iPrevent; patients also completed measures of BC worry, anxiety, risk perception, and knowledge pre- and 2 weeks post-iPrevent. Data were summarized using descriptive statistics.

**Results:**

The SUS and Acceptability questionnaires were completed by 19 of 20 clinicians and 37 of 43 patients. Usability was above average (SUS score >68) for 68% (13/19) clinicians and 76% (28/37) patients. The amount of information provided by iPrevent was reported as “about right” by 89% (17/19) clinicians and 89% (33/37) patients and 95% (18/19) and 97% (36/37), respectively, would recommend iPrevent to others, although 53% (10/19) clinicians and 27% (10/37) patients found it too long. Exploratory analyses suggested that iPrevent could improve risk perception, decrease frequency of BC worry, and enhance BC prevention knowledge without changing state anxiety.

**Conclusions:**

The iPrevent prototype demonstrated good usability and acceptability. Because concerns about length could be an implementation barrier, data entry has been abbreviated in the publicly available version of iPrevent.

## Introduction

Breast cancer (BC) is a major public health problem, accounting for over 2 million cases worldwide each year [1]. In addition to population-based educational and public health policy interventions to minimize exposure to modifiable BC risk factors and optimize cancer screening, identifying women at increased risk and implementing risk-stratified, evidence-based prevention and intensified screening strategies for them is a priority [2]. Health care providers often have difficulty assessing and communicating BC risk as well as the absolute benefits and disadvantages of risk management interventions such as risk-reducing medication, surgery, and cancer screening [3,4].

Several tools exist to estimate BC risk based on personal risk factors, but none provides risk-adapted, individually-tailored, risk management information [5,6]. iPrevent was designed to help women and their health care providers, including primary care physicians (PCP), breast surgeons (BS), and genetics clinicians (GC), to assess and manage BC risk collaboratively [7]. It integrates BC risk estimation, using either the International Breast Cancer Intervention Study model or the Breast and Ovarian Analysis of Disease Incidence and Carrier Estimation Algorithm model (as appropriate for the woman’s risk factors), with tailored risk management information [8-10].

iPrevent users are first given a qualitative risk estimate according to Cancer Australia definitions: average or slightly above average risk (<1.5 times population risk at that age), moderately increased risk (1.5-3 times population risk), or high risk (>3 times population risk) [11]. Women can then choose to see their risk information displayed as a percentage, a pictogram, and a graph. Women are also provided with a menu of risk management strategies appropriate to their risk category, based on Australian National Guidelines [11], with more detailed optional information about each strategy, including estimates of the absolute (rather than relative) risk reductions for each medical and surgical intervention and tailored lifestyle advice.

The aims of this pilot study of patients and their clinicians were to assess the iPrevent prototype with regard to its clinical usability and the acceptability of its content and layout and to identify potential barriers to its implementation. Exploratory aims included assessing its potential impact on patient risk perception, anxiety, BC worry, and BC prevention knowledge.

## Methods

### Study Setting

Stage 1 piloting was undertaken by the researchers with women who had previously received risk assessment and risk management advice at the Peter MacCallum Cancer Centre (PMCC) Breast and Ovarian Cancer Risk Management Clinic [12]. Stage 2 piloting involved PCP, BS, and GC in public hospitals and private primary care and breast and genetics clinics as well as their patients. Patients and clinicians were not selected according to their level of BC risk or prior experience with BC risk assessment.

### Eligibility Criteria

Eligible patients were women aged 18-70 years with no personal history of cancer who provided written informed consent. Patients with previous risk-reducing bilateral mastectomy or major medical comorbidities were excluded. Eligible clinicians were PCP, BS, or GC with a workplace computer with Web access. English proficiency was required for all participants. This study was approved by the Human Research and Ethics Committees of the University of Melbourne and the PMCC. All procedures performed in studies involving human participants were in accordance with the ethical standards of the institutional and/or national research committee and with the 1964 Helsinki declaration and its later amendments or comparable ethical standards.

### Stage 1: Piloting on Patients With Prior Risk Assessment

We enrolled 10 patients from the PMCC Breast and Ovarian Cancer Risk Management Clinic. Baseline information on age, education, computer literacy [13], and both the perceived BC risk category (average, somewhat increased, or substantially increased) [11] and perceived percentage lifetime BC risk were collected. Patients then used iPrevent under the supervision of a research assistant (PW or ES). The time for data input was recorded. Patients were emailed the report in a PDF format. Two weeks after using iPrevent, they completed a questionnaire assessing usability and acceptability of iPrevent, knowledge, and psychosocial outcomes. They could review the emailed iPrevent output while answering these questions.

#### System Usability Scale

This 10-item instrument [14] uses a 5-point Likert rating scale from “strongly agree” to “strongly disagree” to measure product usability. It is applicable to small samples [15] and correlates well with other subjective measures of usability [16,17]. Final scores range from 0-100, and a System Usability Scale (SUS) score >68 is considered above average.

#### iPrevent Acceptability Questionnaire

This 9-item measure, adapted from a previous evaluation of a decision aid [18], uses Likert scales to elicit perceptions of the length, clarity, balance, and usefulness of iPrevent.

#### Breast Cancer Risk Perception

This single item, adapted from a study measuring the impact of genetic counseling, asks patients about their BC risk category: “average,” “somewhat increased,” or “substantially increased” [19]. Women were classified as underestimators, accurate estimators, or overestimators based on comparison with the risk estimated by iPrevent.

#### Breast Cancer Worry Scale

The Lerman BC worry scale is a 3-item scale. Higher scores indicate increased frequency and impact of worry [20].

#### Spielberger State-Trait Anxiety Inventory

The short form State-Trait Anxiety Inventory (STAI; 6 items) measures state anxiety; higher scores indicate higher anxiety [21].

#### Breast Cancer Prevention Knowledge

We used 16 items assessing knowledge regarding BC (11 items), risk-reducing medication (3 items), and risk-reducing mastectomy (2 items), which were adapted from published knowledge measures (see Multimedia Appendix 1) [22,23]. Although every woman was asked to answer all questions, the number of responses scored for each participant was dependent on the iPrevent-determined risk category. All average-risk women and moderate-risk women who were aged <35 years were assessed only on BC knowledge questions. Older, moderate-risk women were also assessed on risk-reducing medication questions. High-risk women were assessed on all 16 questions. The proportion of correct responses was calculated.

### Stage 2: Piloting With Clinicians and Their Patients

We recruited 20 clinicians from previous focus groups [3-4] (5 BS and 3 PCP), via email invitation from KAP (1 BS and 6 GC), and through the PMCC PCP liaison officer (5 PCP). Clinicians first underwent an iPrevent “familiarization” session. Supervised by a research assistant (PW or ES), clinicians first entered data into iPrevent on 3 hypothetical patients (high, moderate, and average risk) and reviewed the iPrevent output information. On the same day, clinicians then conducted 2 mock consultations with female actors: one at high risk and the other moderate risk. Patient (actor) information was pre-entered into iPrevent, and clinicians were asked to use the iPrevent output with the actors as they might in a clinical consultation.

Clinicians were then asked to invite 3 eligible patients from their practice (either during patient appointments or via telephone prior) during the following 3 months to participate by entering their information into iPrevent prior to a consultation and attending an appointment with the clinician to receive the “output.” Patients were provided a printout of their iPrevent output via email. Clinicians recorded the amount of time spent using iPrevent.

All patients were asked to complete the same pre- and post-iPrevent assessments as in Stage 1. Clinicians completed the SUS and Acceptability questionnaires 2 weeks after recruitment of 3 patients (or 3 months after familiarization, if full patient recruitment did not occur).

### Statistical Analyses

All statistical analyses were performed in R 3.2.3 (R Core Team, 2015). The planned sample size of 20 clinicians and 60 patients was based on pragmatic estimates of the numbers it was considered possible to recruit over the available time period. The purpose of the study was to assess the acceptability and usability of iPrevent for clinicians and patients and not to test hypotheses. Therefore, descriptive statistics were used to summarize the data (mean, median, and range for continuous variables and counts and percentages for categorical variables). Patient and clinician data were analyzed separately. A pairwise *t* test was used to assess whether the STAI score changed from pre- to post-iPrevent assessment.

## Results

### Participants

We recruited 20 clinicians and 43 patients (10 for Stage 1 and 33 for Stage 2). Clinicians only recruited 33 of the planned 60 patients (planned 3 per clinician). BS (n=6) recruited 16 of a planned 18 patients, GC (n=6) recruited 14 of a planned 18 patients (1 GC moved overseas during the study and was, thus, unable to recruit her 3 planned patients), and PCP (n=8) recruited only 3 of a planned 28 patients.

### Participant Characteristics

Patient characteristics are shown in Table 1. Median age was 38 years (range 21-56 years), 74% (31/42) had a university education, and 51% (22/43 were at moderate risk for BC. Clinician characteristics are shown in Table 2. Their median age was 47 years (range 28-66 years); of all clinicians, 40% (8/20) were PCP, 30% (6/20) were BS, and all but 15% (3/20) were females. The majority used computers often and rated themselves as having good computer skills.

### iPrevent Data Entry and Consultation Times

Patients took a median of 15 (range 5-60) minutes to enter their risk factor data. The median time taken for clinician consultations in which iPrevent data were discussed was 20 (range 5-45) minutes.

### System Usability Scale

**Figure 1:**
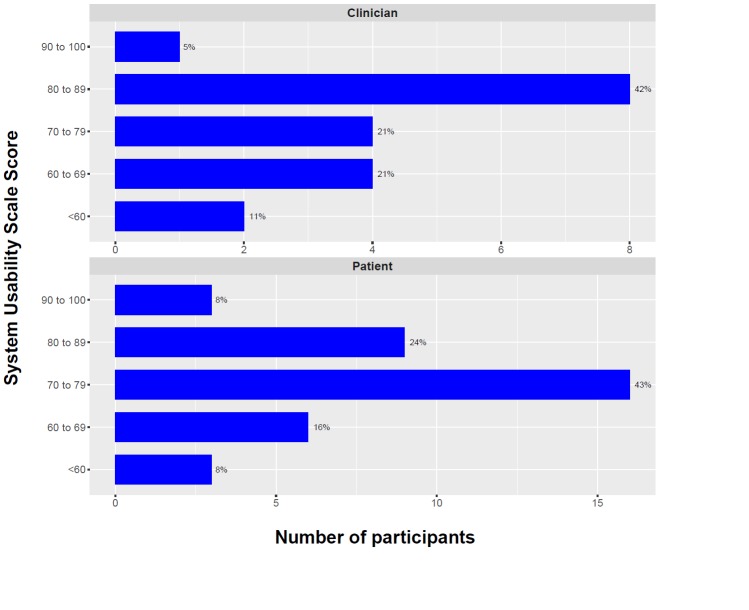
iPrevent System Usability Scale scores for clinicians and patients.

SUS responses are summarized in Figure 1. Data were missing for 6 patients and 1 clinician who did not return the questionnaire. Overall, 76% (28/37) patients and 68% (13/19) clinicians rated iPrevent usability as above average (SUS score >68).

**Table 1:** Patient characteristics (N=43).

Characteristic		Value
Age in years^a^, median (range)		38 (21-56)
**Highest level of education, n (%)^a^**		
	Secondary school	6 (14)
	Vocational training	5 (12)
	University	31 (74)
**Use of computers at work or elsewhere, n (%)^a^**		
	Often	33 (79)
	Sometimes	8 (19)
	Rarely	1 (2)
**Computer skills (self-reported), n (%)^a^**		
	Expert	9 (21)
	Good	29 (69)
	Poor	4 (10)
**Sources of information on breast cancer (BC) risk in the past, n (%)^a^**		
	Health professional	30 (71)
	Family and friends	19 (45)
	Internet	10 (24)
	Health information booklets	6 (14)
	Support organizations	4 (10)
**BC risk category estimated by iPrevent, n (%)**		
	Average	14 (33)
	Moderate	22 (51)
	High	7 (16)
“**Do you feel like you know what your own risk of breast cancer is?” n (%)^b^**		
	Don’t know my risk	12 (29)
	I think I know my risk	25 (61)
	Confident I know my risk	4 (10)

^a^Data missing for1 patient.

^b^Data missing for 2 patients.

**Table 2:** Clinician characteristics (N=20).

Characteristic			Value
Age in years, median (range)			47 (28-66)
**Gender, n (%)**			
	Female	17 (85)	
	Male	3 (15)	
**Year of graduation, n (%)**			
	1970-1979	1 (5)	
	1980-1989	7 (35)	
	1990-1999	4 (20)	
	2000-2009	7 (35)	
	2010-2015	1 (5)	
**Specialty, n (%)**			
	Breast surgeon	6 (30)	
	Genetic counselor	3 (15)	
	Geneticist	2 (10)	
	Primary care physician	8 (40)	
	Medical oncologist	1 (5)	
Number of years working in specialty, median (range)			16 (1–34)
How many patients per year would you discuss breast cancer risk with? Median (range)			138 (4–960)
**Use of computers at work or elsewhere, n (%)**			
	Often	20 (100)	
	Sometimes	0 (0)	
	Rarely	0 (0)	
**Computer skills (self-reported), n (%)**			
	Expert	0 (0)	
	Good	18 (90)	
	Poor	2 (10)	

**Table 3:** iPrevent acceptability among clinicians and patients.

Acceptability assessment		Clinician, n (%)	Patient, n (%)
**The amount of information provided is^**a,b**^**			
	Too much	0 (0)	1 (3)
	A little too much	2 (11)	3 (8)
	About right	17 (89)	33 (89)
**The length of the tool is^**a,b**^**			
	Much too long	4 (21)	0 (0)
	A little too long	6 (32)	10 (27)
	About right	9 (47)	27 (73)
**Clarity of information^**a,b**^**			
	Very clear	8 (42)	7 (19)
	Mostly clear	5 (26)	16 (43)
	About right	5 (26)	13 (35)
	Not clear	1 (5)	1 (3)
**Regarding cancer prevention, how balanced did the information seem^**b,c**^**			
	Biased toward prevention	3 (17)	9 (24)
	Completely balanced	14 (78)	26 (70)
	Biased against prevention	1 (6)	2 (5)
**Any of the information new to you^**a,b**^**			
	All	0 (0)	1 (3)
	Most	0 (0)	11 (30)
	Some	12 (63)	22 (59)
	None	7 (37)	3 (8)
**How helpful with regard to making a decision about BC risk management^**a,b**^**			
	Very helpful	9 (47)	19 (51)
	Somewhat helpful	7 (37)	13 (35)
	A little helpful	3 (16)	5 (14)
**Recommend this tool to others^**a,b**^**			
	Definitely	12 (63)	18 (49)
	Probably	6 (32)	18 (49)
	Probably not	1 (5)	1 (3)
**How simple to navigate through the tool^**a,b**^**			
	Very easy	7 (37)	21 (57)
	Somewhat easy	11 (58)	15 (41)
	Not easy	1 (5)	1 (3)
**Easy to read^**a,b**^**			
	Very easy	8 (42)	22 (59)
	Somewhat easy	11 (58)	15 (41)

^a^n=19 clinicians because of missing data for 1 clinician.

^b^n=37 patients because of missing data for 6 patients.

^c^n=18 clinicians because of missing data for 2 clinicians.

### iPrevent Acceptability Questionnaire

Table 3 shows that iPrevent was generally acceptable to study participants. Of all, 89% (17/19) clinicians and 89% (33/37) patients reported that the amount of information provided by iPrevent was “about right.” Furthermore, 53% (10/19) clinicians and 27% (10/37) patients reported that iPrevent was too long. Only 1 patient and 1 clinician reported that the information was not clear and that they would “probably not” recommend iPrevent to others.

### Exploratory Endpoints

#### Breast Cancer Risk Perception

Of patients who completed the relevant questions before iPrevent, 40% (14/35) correctly indicated their BC risk category, but 51% (18/35) overestimated and 9% (3/35) underestimated their BC risk category. Post-iPrevent, 86% (30/35) accurately estimated their risk category, although 11% (4/35) and 3% (1/35) continued to overestimate and underestimate their risk, respectively.

#### Breast Cancer Worry Scale

Pre-iPrevent, 26% (11/42) women reported worrying about BC “often” or “all the time,” while 19% (7/37) women reported this after iPrevent. Regarding the impact of BC worry on mood and daily activities, 69% (29/42) patients reported a low score (1-1.5 out of 4) pre-iPrevent. When this was compared before and after iPrevent, 25% (9/36) patients reported less impact, 47% (17/36) reported no change, and 28% (10/36) reported more impact.

#### Spielberger State-Trait Anxiety Inventory

The mean short form STAI score (maximum 24) pre-iPrevent was 11.3 (SD 3.8) with no significant change post-iPrevent (median increase of 1, 95% CI: 0.5-2; *P*=.14).

### Breast Cancer Prevention Knowledge

Overall BC prevention knowledge improved for all risk groups. (Multimedia Appendices 1 and 2).

## Discussion

This pilot study of the iPrevent prototype has found good usability and acceptability without evidence of an adverse impact on anxiety or BC worry. The observation that the 8 PCP recruited only 3 patients between them in the required 3-month period suggests that implementation of iPrevent into primary care might be substantially more challenging than implementation into the specialist setting, where recruitment of patients was much higher. Another interpretation is that the study requirements (eg, obtaining written informed consent) were onerous, especially for PCP in busy practices, and thus, the low recruitment by PCP in this study might not reflect the uptake of iPrevent in routine practice. However, as earlier focus groups had highlighted that PCP generally do not see BC risk assessment and management as being in their domain, iPrevent might be able to contribute to overcoming provider unfamiliarity and lack of confidence for this group of clinicians [3].

The prototype was considered too long by a majority of clinicians and some patients, indicating another potential barrier to implementation. Patients took a median of 15 minutes and up to 60 minutes to enter their risk factor data, and the subsequent median time taken for the clinician consultation using the iPrevent output was 20 minutes. To address this issue, we have now incorporated changes to streamline the data entry for family history. This study also highlighted the need for patients to be able to enter their data into iPrevent at home prior to a consultation.

iPrevent may improve BC risk perception given an additional 46% (16/35) patients accurately estimated their BC risk category after using iPrevent. As a higher perceived risk of BC is associated with considering medical prevention and risk-reducing surgery among high-risk women [24-26], iPrevent could become a potential behavior-modifying tool. While this pilot study provides no information about the uptake of risk management strategies after using iPrevent, this issue will be an important endpoint for future larger studies. Other studies have found that women who have access to more thorough information from genetic counselors, combined with support to make decisions, have a higher uptake of risk reduction methods [27-29]; thus, we hypothesize that iPrevent might have a similar impact.

Use of iPrevent did not appear to increase patient worry or anxiety, consistent with the literature that has found that decreased anxiety and better psychological outcomes are associated with improved accuracy of perceived risk [24,30,31]. Use of iPrevent seemed to improve BC knowledge, a recognized critical first step in helping individuals understand screening options, weigh potential benefits and risks for risk-reducing measures, and make informed decisions [32-34]. In addition, 89% (33/37) patients indicated that some or most of the information contained in iPrevent was new to them (Table 3).

This pilot had several limitations. First, the sample was small and the study did not achieve its target patient recruitment. The majority of patients were young and highly educated, so the acceptability and usability of iPrevent might differ in the general community where computer literacy might be lower. Similarly, clinicians who chose to participate could have been more highly engaged with BC risk assessment and risk management than nonparticipant clinicians. Finally, only short-term outcomes were measured, and the impact on long-term satisfaction and uptake of BC risk-reducing measures could not be determined. As a result of this study, enhancements have been made to iPrevent with the aim of further increasing acceptability and usability.

## Appendix

Multimedia Appendix 1 Breast Cancer Prevention Knowledge Questionnaire.

Multimedia Appendix 2 Results of Pre- and Post-iPrevent® Knowledge Questionnaire.

## Abbreviations

BC: breast cancer
BS: breast surgeon(s)
GC: genetics clinician(s)
PCP: primary care physician(s)
PMCC: Peter MacCallum Cancer Centre
STAI: State-Trait Anxiety Inventory
SUS: System Usability Score
