# Supplementary material for: The iPrevent Online Breast Cancer Risk Assessment and Risk Management Tool: Usability and Acceptability Testing
Source: JMIR Form Res. 2018 Nov 7;2(2):e24. doi: 10.2196/formative.9935 (PMC6334700; doi:10.2196/formative.9935)
Supplement: Multimedia Appendix 1 [file formative_v2i2e24_app1.pdf]

Appendix 1: Breast Cancer Prevention Knowledge Questionnaire

**The next 16 questions ask you about breast cancer risk factors and screening.** **Please indicate for each item whether it is True, False, or if you Don't know.**	
1. Regular moderate exercise does not reduce your risk of breast cancer	☐ True ☐ False ☐ Don't know
2. Maintaining a healthy weight is important for minimising breast cancer risk	☐ True ☐ False ☐ Don't know
3. You should promptly report any new or unusual changes in your breasts to your GP	☐ True ☐ False ☐ Don't know
4. Screening mammograms are recommended every two years for women aged 50-74 years	☐ True ☐ False ☐ Don't know
5. Using hormone replacement therapy (HRT) may decrease your risk of breast cancer	☐ True ☐ False ☐ Don't know
6. About 1% (10 in 1000) of Australian women will develop breast cancer in their lifetime	☐ True ☐ False ☐ Don't know
7. Consuming alcohol can decrease your risk of breast cancer	☐ True ☐ False ☐ Don't know
8. The more children you have, and the earlier you have them, the lower your risk of breast cancer	☐ True ☐ False ☐ Don't know
9. Breast feeding can increase your risk of breast cancer	☐ True ☐ False ☐ Don't know
10. Mammograms reduce your risk of developing breast cancer	☐ True ☐ False ☐ Don't know
11. Medications such as Tamoxifen may reduce your risk of breast cancer	☐ True ☐ False ☐ Don't know
12. Medications such as Tamoxifen are recommended to be taken for 1 year	☐ True ☐ False ☐ Don't know

13. Taking medications such as Tamoxifen is not recommended for women with a history of blood clots or those who smoke	☐ True ☐ False ☐ Don’t know
14. Your breast cancer risk can be slightly increased while you are using a hormonal contraceptive, such as the contraceptive pill	☐ True ☐ False ☐ Don’t know
15. Risk-reducing mastectomy means removing as much breast tissue as possible before a cancer develops	☐ True ☐ False ☐ Don’t know
16. A woman who has her breasts removed will definitely not develop breast cancer	☐ True ☐ False ☐ Don’t know
