# Supplementary material for: The iPrevent Online Breast Cancer Risk Assessment and Risk Management Tool: Usability and Acceptability Testing
Source: JMIR Form Res. 2018 Nov 7;2(2):e24. doi: 10.2196/formative.9935 (PMC6334700; doi:10.2196/formative.9935)
Supplement: Multimedia Appendix 2 [file formative_v2i2e24_app2.pdf]

Appendix 2: Results of Pre- and Post-iPrevent® Knowledge Questionnaire

**Risk Category**	**No. of women**	**Pre-iPrevent® mean % questions correct [range]**	**Post- iPrevent® mean % questions correct [range]**
Average and Moderate < 35yrs[a] (11 questions)	17	69% [45-91%]	77% [45-91%]
Moderate > 35yrs (14 questions)	15	54% [7-86%]	76% [29-100%]
High (16 questions)	4	89% [75-100%]	92% [88-94%]

[a] 5 moderate risk women not shown risk reducing medication information as <35 yrs old hence only assessed on 11 general knowledge questions
